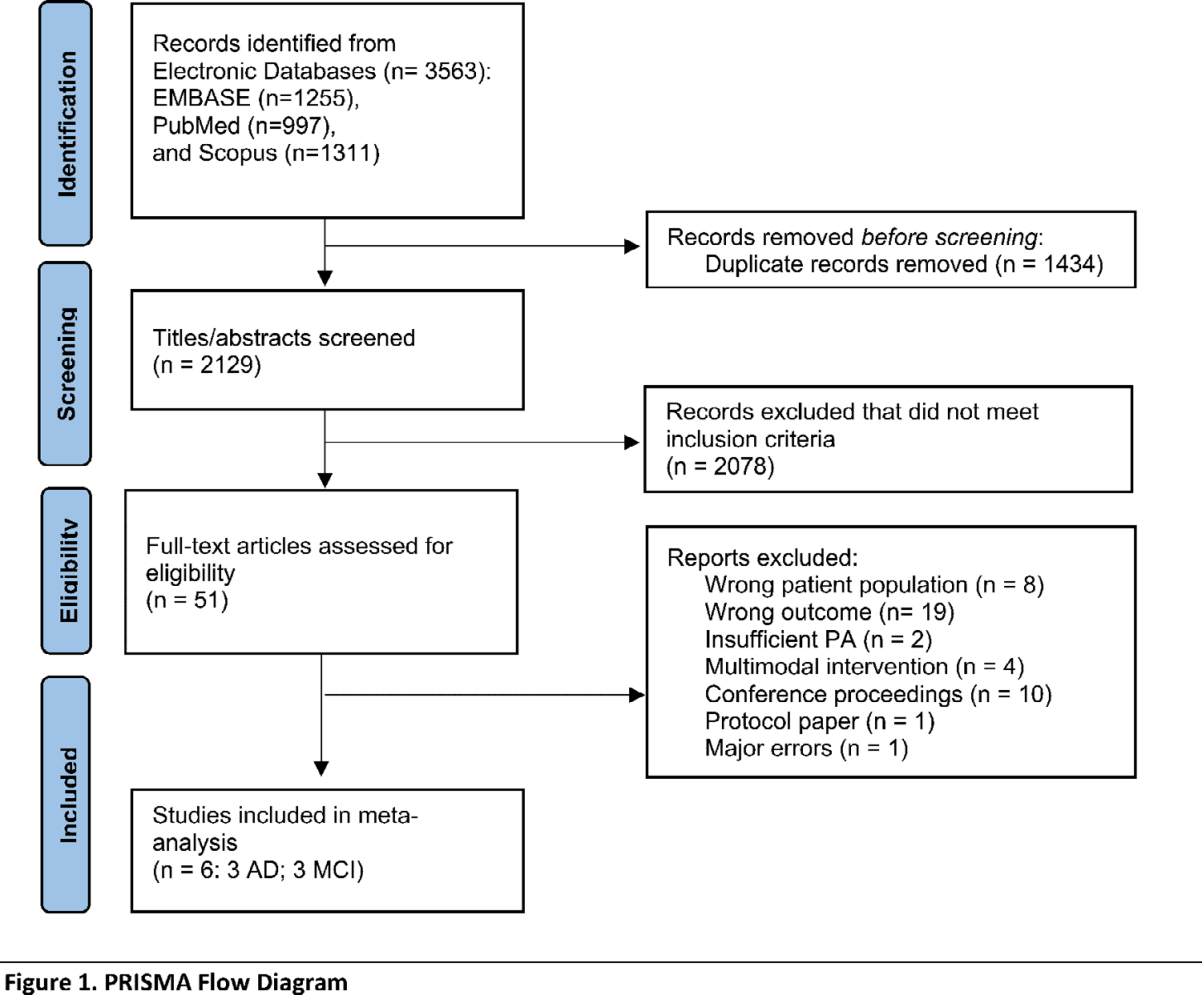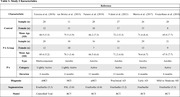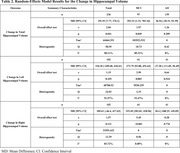# The Impact of Physical Exercise on Hippocampal Atrophy in Mild Cognitive Impairment and Alzheimer’s Disease: A Meta‐analysis in Support of Early Detection and Intervention

**DOI:** 10.1002/alz.093139

**Published:** 2025-01-09

**Authors:** Gavin T Kress, Emily S. Popa, David A. Merrill, Jennifer E. Bramen, Prabha Siddarth

**Affiliations:** ^1^ The Icahn School of Medicine at Mount Sinai, New York, NY USA; ^2^ Pacific Brain Health Center, Pacific Neuroscience Institute and Foundation, Santa Monica, CA USA; ^3^ David Geffen School of Medicine at University of California Los Angeles, Los Angeles, CA USA; ^4^ Saint John’s Cancer Institute at Providence Saint John’s Health Center, Santa Monica, CA USA

## Abstract

**Background:**

Alzheimer’s disease (AD) presents a significant global health challenge, with limited pharmacological interventions available. Physical activity (PA) is a promising therapeutic approach for delaying AD onset and mitigating neuropathology. Only a handful of meta‐analyses have explored the effects of exercise interventions on structural MRI (sMRI)‐estimated brain volumes in adults with mixed cognitive statuses, two of which found positive effects on left and bilateral hippocampal volume (HV). No meta‐analysis to date has solely included studies examining the effect of PA on sMRI outcomes in individuals with cognitive impairment (CI). In this meta‐analysis, we examined whether there is evidence to support the hypothesis that PA interventions positively impact HV in individuals with CI. We also assessed whether the level of CI (Mild Cognitive Impairment (MCI) vs. AD) impacted this relationship.

**Method:**

We identified six controlled trials that investigated effects of PA interventions on HV in older individuals with cognitive impairment and met inclusion criteria **(Table 1)**. These included 236 participants with AD, MCI, or preclinical AD. Data were extracted and analyzed following Cochrane guidelines **(Figure 1)**. We used a random‐effects model (PythonMeta library: Python 3.9) to estimate the mean change in HV pre‐ and post‐exercise intervention. Forest plots, Hedges’ g funnel plots, and Egger’s test were used to assess unbiasedness and visualize intervention effects, and Tau^2^, Cochran’s Q, and I^2^ were calculated to assess heterogeneity.

**Result:**

The primary analysis revealed a significant positive effect of exercise on total hippocampal volume (p = .04). However, sub‐group analyses indicated a significant preservation of HV only in individuals with MCI (**Table 2**; Bilateral: p = 0.05; L: p = 0.003; R: p<0.001), but not in those with AD (Bilateral: p = 0.2; L: p = 0.5; R: p = 0.8). Egger’s test indicated no evidence of publication bias (p = 0.3). Subgroup analyses also revealed significant heterogeneity only within the MCI cohort for the total and left HV.

**Conclusion:**

Exercise interventions demonstrated a moderate, significant effect in preserving HV among individuals with MCI, but not AD, highlighting a potential therapeutic benefit, particularly in earlier disease stages. These findings encourage future investigation of PA as a treatment for MCI and further incentivize earlier detection.